# Jaborandi

**Published:** 1875-12

**Authors:** 


Jaborandi.—A note in the New York *Medical Journal* states that, at Bellevue Hospital, Dr. Janeway has been using jaborandi, but has not been able to get as satisfactory effects as have been reported by some observers. One drachm of the powder was given in the form of an infusion of four ounces, and at the end of half an hour there was an increase of the temperature of $\frac{1}{2}^{\circ}$, with slight tendency to diaphoresis. In the second case, one drachm was given mixed with water, and again no result followed, beyond an increase in the frequency of the pulse. In the third case, Dr. Knox, the house physician, took one drachm mixed with water. In forty-five minutes marked salivation occurred, but no diaphoresis.—*Med. and Surg. Reporter.*

